# The relationship between sleep quality and occupational well-being in employees: The mediating role of occupational self-efficacy

**DOI:** 10.3389/fpsyg.2023.1071232

**Published:** 2023-01-27

**Authors:** Jiaxi Peng, Jiaxi Zhang, Bingbing Wang, Yanchen He, Qiuying Lin, Peng Fang, Shengjun Wu

**Affiliations:** ^1^Mental Health Education Center & College of Teachers, Chengdu University, Chengdu, China; ^2^Xi’an Research Institute of High-Technology, Xi’an, China; ^3^Aviation University of Air Force, Changchun, China; ^4^Department of Military Medical Psychology, Air Force Medical University, Xi’an, Shaanxi, China

**Keywords:** employees, occupational self-efficacy, occupational well-being, positive psychology, sleep quality

## Abstract

**Objective:**

This study aimed to examine the impact of sleep quality on occupational well-being in employees by primarily focusing on the mediating role of occupational self-efficacy.

**Methods:**

A total of 487 junior staff completed a set of questionnaires comprised Pittsburgh Sleep Quality Index scale, Occupational Self-efficacy Scale, and occupational well-being measurements.

**Results:**

The results revealed that both sleep quality and occupational self-efficacy were significantly correlated with occupational well-being. The structural equation modeling analysis and the bootstrap test indicated that occupational self-efficacy partially mediated the effect of poor sleep quality on occupational well-being.

**Discussion:**

These findings expand upon existing research on the relationship between sleep quality and well-being among occupational workers, shed light on the correlation of poor sleep quality with occupational well-being, and are valuable in promoting the occupational well-being of employees.

## Introduction

Sleep is a fundamental biological process that is required for the survival and development of humans (Darchia et al., 2018; Agostini et al., 2019; Matricciani et al., 2019; Jean-Louis et al., 2020). A lack of quality sleep induces body discomfort, psychological imbalance, physical diseases, and mental disorders (Tanaka and Shirakawa, 2004; Cheng et al., 2017; Guglielmi et al., 2018). Sleep insufficiency and low sleep quality are common problems faced by a variety of populations for multiple reasons; hence, sleep problems have become a serious and widespread challenge in modern societies worldwide (Perlus et al., 2018; Bagheri Hosseinabadi et al., 2019). These problems are more common among working people (Skarpsno et al., 2018). Investigations have shown that an average daily sleep duration of less than 6 h was 29.9% among American employees and up to 40.5% among managers and entrepreneurs; additionally, total sleep time has declined over the last decade (Luckhaupt et al., 2010; Welsh et al., 2014). In 2015, sleep disorders were recognized as a “public health epidemic disease” by the US Center for Disease Control and Prevention, and this heralded subsequent attempts to largely promote education on “sleep health” (Faber et al., 2017). Similarly, this problem has received wide attention in nations such as the United Kingdom, Finland, Sweden, and South Korea (Groeger et al., 2004; Salminen et al., 2010). *The Sleep Index Report of China* demonstrated that the rate of low sleep quality among Chinese residents in 2015 was 31.2, and 37.8% of people in the workplace suffer from sleep problems which were found to affect the daytime work efficiency (Shi and Long, 2018). *The 2016 Internet Employee Sleep Report of China* showed that up to 81.4% of Internet-based workers had low sleep quality (Lin et al., 2018).

Given the reality of sleep quality all around the world, and that low sleep quality impairs the psychological and physical health of people (Tanaka and Shirakawa, 2004; Weaver et al., 2018; Barnes and Watson, 2019), a better understanding of the implications of poor sleep quality for people’s daily working and state of life is required. Specifically, poor sleep of employees has often induced many negative consequences, such as a decline in in-role performance, poor interpersonal relationships in the workplace, a decline in organizational citizenship behaviors, and an increase in unethical workshop behaviors, thereby further negatively affecting the routine work and orderly operation of various organizations (Barnes et al., 2011; Lanaj et al., 2014; Bolino and Klotz, 2015; Barnes and Watson, 2019). Hence, sleep problems and their effects on the workplace environment comprise a research theme with practical significance.

### Sleep quality and subjective well-being

Reportedly, poor sleep quality has often been related to negative affect and when sustained with time was a risk factor for affective disorders (Nicassio et al., 2012; Kao et al., 2016; Weinberg et al., 2016). Biological evidence indicated that this relationship would exist because of the mutual relation with the serotonergic system, which is hypothesized to play a considerable role in the regulation of sleep and affect (Welsh et al., 2014; Weinberg et al., 2016). Given those close relationships, sleep quality may have significant implications for other affectively driven variables, such as subjective well-being. The pursuit of well-being by individuals is considered an everlasting theme throughout the lifespan with a strong power to be used constructively for social development (He et al., 2014). Along with the emergence of positive psychology, various studies have been conducted on subjective well-being. Subjective well-being involves overall cognitive (satisfaction with life) and affective (positive or negative) evaluations of a person’s life (Zhang et al., 2014a). Sleep quality is closely related to subjective well-being (Tighe et al., 2016). Low sleep quality causes a decrease in satisfaction with life, thereby leading to a reduction in positive affect and an increase in negative affect (Berger et al., 2012; Palmer and Alfano, 2017; Kim, 2018). For example, Weinberg et al. (2016) documented that sleep quality was positively correlated with subjective well-being and partially mediated the relationship between stress and subjective well-being. Hamilton et al. (2007) found that insomnia was significantly correlated with well-being, whereby individuals with insomnia symptoms had lower psychological and subjective well-being. Espie et al. (2019) showed that following cognitive behavioral therapy, the sleep quality of insomniac individuals significantly improved, and this was accompanied by significant improvements in well-being. In all, from the existing research, it can be obtained that poor sleep quality leads to lower satisfaction with life, less positive affect, and more negative affect.

### Sleep quality and occupational well-being

Based on the circumflex model of affect and the hedonistic orientation of well-being, Bakker and Oerlemans (2011) defined occupational well-being as the cognitive appraisal and emotional experience in the workplace setting, which refers to general satisfaction with one’s job and their experiences of positive or negative emotions at work. As previously mentioned, the relationship between sleep and subjective well-being has been extensively investigated. However, studies on the association between sleep quality and occupational well-being are limited. Based on previous findings, it is reasonably hypothesized that sleep quality is related to occupational well-being. The reasoning for this is as follows. Firstly, occupational well-being refers to an individual’s satisfaction with their job, and is also part of their overall satisfaction with life, namely, subjective well-being (Bakker and Oerlemans, 2011; Bretones and Gonzalez, 2011; Wang and Peng, 2017). Hence, the relationship between sleep and occupational well-being may be similar to that between sleep and subjective well-being. Secondly, occupational well-being consists of job satisfaction and both positive and negative emotions at work (Bakker and Oerlemans, 2011). Many studies have demonstrated that sleep is related to job satisfaction and work emotion. For example, Scott and Judge (2006) found that insomnia was associated with increased feelings of hostility and decreased feelings of joviality and attentiveness, as well as decreased job satisfaction. Lai et al. (2008) found that sleep quality was among the key factors affecting job satisfaction and the turnover intention of nurses. Barnes et al. (2013) documented that sleep quality significantly predicted job satisfaction and the organizational citizenship behaviors of employees. Additionally, low sleep quality has been related to conflicts between leaders and their followers (Guarana and Barnes, 2017), as well as feelings of anxiety, depression, anger, and hostility within the workplace (Granö et al., 2008; Weaver et al., 2018).

On this basis, we proposed hypothesis 1: *Sleep quality is related to the occupational well-being of employees*.

### The mediating role of occupational self-efficacy between sleep quality and occupational well-being

Though much international study has been reported, the mechanism underlying the effect of sleep quality on the occupational well-being of employees has remained unclear. Previous research on the relationship between sleep quality and occupational well-being of employees have used two-valued logic, but it is unknown what role of occupational self-efficacy might play as an intervention in the promotion of occupational well-being through reducing the negative behavioral and psychological symptoms effects of poor sleep quality and increasing the experience of self-control in career (Guarnaccia et al., 2018; Van Hootegem et al., 2022). Thus, the present study focused on occupational self-efficacy, which is a domain-specific self-efficacy that refers to the belief of whether one is competent for tasks and activities at work (Çetin and Aşkun, 2018). Previous studies have documented that sleep quality is related to self-efficacy (Przepiórka et al., 2019). For example, Schlarb et al. (2015) found that college students who reported having frequent nightmares generally also reported lower self-efficacy than their classmates. Furthermore, most studies have held that self-efficacy promotes the mental health and subjective well-being of individuals. For instance, self-efficacy has been suggested to be positively associated with self-esteem, optimism, and satisfaction with one’s life and job, while being negatively associated with feelings of anxiety, depression, and fatigue (Strobel et al., 2011; Peng et al., 2018; Ngui and Lay, 2020). Bandura (1993) held that because conducted activities are different between different domains, they require different abilities and skills, and hence, self-efficacy toward various tasks differs. Self-efficacy is always related to specific domains. Compared with general self-efficacy, domain-specific self-efficacy has been found to better predict cognitive abilities and behaviors in specific domains (Paunonen and Hong, 2010; Grether et al., 2018). In the occupational environment, self-efficacy and well-being could be specific to occupational self-efficacy and occupational well-being. Therefore, it is logical to hypothesize that there exist relationships between sleep quality, occupational self-efficacy, and subjective well-being.

With this, we put forward hypothesis 2: *Occupational self-efficacy mediates the relationship between sleep quality and occupational well-being*. In other words, the belief of employees that they are competent for relevant tasks might play as a mediating role in the promotion of occupational well-being (increase job satisfaction but less negative emotions at work) through reducing the negative effects of low sleep quality and increasing the belief of an individual’s ability to meet their job requirements.

### Aim of the present study

We aimed to expand upon existing research on the relationship between sleep quality and occupational well-being among employees. Furthermore, we explored how sleep quality affects occupational well-being and verified the mediating role of occupational self-efficacy in the relationship between sleep quality and occupational well-being.

## Materials and methods

### Participants and procedure

The participants of this study were 487 working adults who came from several firms based in Chengdu, China. Their domains of the work included decoration, food, environmental protection, and logistics transportation. All managers, sleep disorder, and shift workers were excluded. The participants included 309 male and 178 female, and ranged in age from 24 to 46 years, with a mean age of 29.74 years (standard deviation = 8.49). A total of 274 (63%) of the participants had received a bachelor’s degree or above. The average duration of their employment was 37.43 months. All participants were administered questionnaires between December 2020 and February 2021.

Before the investigation, the researchers explained study’s purpose and ethical aspects, all participants read and signed the provided informed consent form. The paper- and pencil questionnaires were administered in the classroom environment, and all subjects answered anonymously. In total, 487 copies of the questionnaire were distributed to the participants and 433 valid copies were returned. All participants voluntarily took part in this survey and were given 10 RMB (about 1.5 US dollars) for their participation. The research described in this paper met the standards of the author’s university and has been approved by its ethics committee.

### Instruments

#### Pittsburgh sleep quality index

The Pittsburgh Sleep Quality Index (PSQI) is a 19-item self-rated questionnaire used for evaluating subjective sleep quality over the previous month. Some example questions include “how long (in minutes) has it usually taken for you to fall asleep each night?” and “how many hours of actual sleep did you get at night?.” The 19 questions were combined into 7 clinically-derived components, with each weighted equally from 0 to 3. The seven component scores were added to obtain a global score ranging from 0 to 21, with higher scores indicating worse sleep quality, and a total score > 7 indicates sleep disorders (Carpenter and Andrykowski, 1998). The PSQI was translated into the Chinese language and showed good reliability and validity (Tsai et al., 2005). The Cronbach’s alpha coefficient of the PSQI in the present study was 0.73.

#### Occupational self-efficacy scale

The Occupational Self-efficacy Scale (OSS), developed by Rigotti et al. (2008), involved six items. Some examples include “I meet the goals that I set for myself in my job” and “I feel prepared for most of the demands in my job.” Participants’ responses were rated using a 6-point scale ranging from 1 = *completely not true* to 6 = *completely true*. The OSS has previously been translated into the Chinese language and used in Chinese studies (Liu, 2019). The Cronbach’s *α* coefficient of the OSS in this study was 0.85.

#### Occupational well-being measurements

The occupational well-being of participants was assessed using three subscales, which evaluated job satisfaction, job-related positive affect, and job-related negative affect, respectively (Dicke et al., 2018). The Michigan job satisfaction scale included three items (e.g., “All in all, I am satisfied with my job”), and each item was rated by a 5-point scale from 1 (strongly disagree) to 5 (strongly agree; Bowling and Hammond, 2008). The positive and negative job-related affect scales were comprised 10 questions each. Each item described one type of positive or negative emotion from work, such as “My job made me feel energetic” and “My job made me feel bored.” The participants were asked to rate how often they experienced these emotional states using a 5-point rating scale from “1” (never) to “5” (extremely often; Van Katwyk et al., 2000). The measurements were previously translated into Chinese for use in past studies (Li et al., 2014). The Cronbach’s alpha coefficient of the three subscales in the present study was 0.87, 0.79, and 0.84, respectively.

### Data analysis

Pearson’s correlation analysis was first used to identify the relationships between sleep quality, occupational self-efficacy, job satisfaction, and job-related positive and negative affect. Then, a series of regression analyses were conducted using job satisfaction and job-related positive and negative affect as dependent variables, with sleep quality as an independent variable. If the regression coefficients of sleep quality were significantly changed after occupational self-efficacy was introduced into the equations, which was used as a criterion to evaluate whether occupational self-efficacy played the mediating role in the relationship between sleep quality and occupational well-being. Finally, occupational well-being was regarded as a latent variable. The structural equation model analysis and the bootstrap test were adopted to test the significance of the indirect and direct effects of sleep quality on occupational well-being.

## Results

Results showed 152 (35.10%) participants have sleep disorders. The Pearson’s correlation analysis involving poor sleep quality, occupational self-efficacy, job satisfaction, and job-related positive and negative affect was conducted (Table 1). Significant correlations were found between any two variables. The results supported hypothesis 1 and confirmed that sleep quality significantly correlated with occupational well-being of employees.

**Table 1:** Descriptive statistics and correlation analysis of all the variable (*n* = 433).

	Mean	SD	1	2	3	4	5
1. Poor sleep quality	6.58	3.54					
2. Occupational self-efficacy	3.91	0.86	0.25^**^				
3. Job satisfaction	3.59	0.91	−0.23^**^	−0.35^**^			
4. Positive affect	3.19	0.79	0.25^**^	0.37^**^	−0.39^**^		
5. Negative affect	2.61	0.57	0.24^**^	0.32^**^	−0.51^**^	0.54^**^	

^**^p < 0.01.

For the purpose of further confirming research hypothesis 2 and exploring the relationship among occupational self-efficacy, poor sleep quality, and occupational well-being, a series of regression analyses were conducted. Table 2 summarizes the regression analysis process. In Model 1, occupational self-efficacy was the dependent variable and poor sleep quality was the independent variable. The analysis showed that poor sleep quality significantly predicted occupational self-efficacy. Then, in models 2–4, the results showed that poor sleep quality significantly predicted job satisfaction, job-related positive affect, and job-related negative affect. In models 5–7, when occupational self-efficacy was introduced into the models as the independent variable together with sleep quality, the results showed that the regression coefficients of poor sleep quality to job satisfaction, job-related positive affect, and job-related negative affect were all decreased.

**Table 2:** Regression analysis (*n* = 433).

Model	Dependent	Predictors	Model summary		Coefficients			
			*F*	*R^2^*	B	SE	β	*t*
1	OSE	Poor sleep quality	41.23^**^	0.09	−0.07	0.01	−0.30	−6.42^**^
2	Job satisfaction	Poor sleep quality	72.26^**^	0.14	−0.10	0.01	−0.38	−8.50^**^
3	Positive affect	Poor sleep quality	39.86^**^	0.08	−0.07	0.01	−0.29	−6.31^**^
4	Negative affect	Poor sleep quality	34.32^**^	0.07	0.04	0.01	0.27	5.86^**^
5	Job satisfaction	Poor sleep quality	68.21^**^	0.24	−0.07	0.01	−0.28	−6.42^**^
		OSE			0.34	0.05	0.33	7.42^**^
6	Positive affect	Poor sleep quality	38.76^**^	0.15	−0.05	0.01	−0.21	−4.53^**^
		OSE			0.25	0.04	0.27	5.88^**^
7	Negative affect	Poor sleep quality	3.42^**^	0.12	0.03	0.01	0.20	1.53^**^
		OSE			−0.16	0.03	−0.23	−4.96^**^

OSE, occupational self-efficacy. **p<0.01.

**Figure 1:**
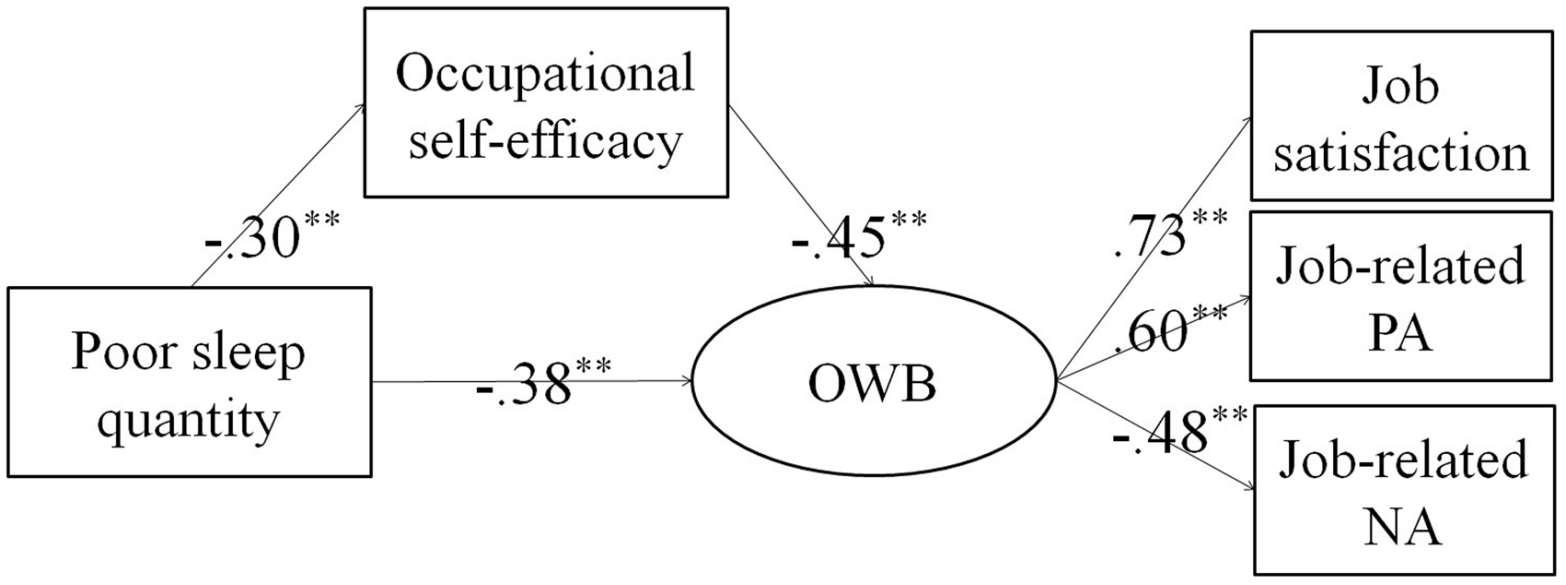
The partial mediating model. All coefficients were standardized. ^**^p < 0.01. OWB, occupational well-being; PA, positive affect; NA, negative affect.

Lastly, structural modeling analyses were used to further explain the relationships between variables. In the first step, the direct effect of the predictor variable (poor sleep quality) on the dependent variable (occupational well-being) without mediators was tested. The directly standardized path coefficient was significant (β = −0.51, *p* < 0.01). We then evaluated a partially mediated model, which contained the mediator (occupational self-efficacy) and a direct path from poor sleep quality to occupational well-being (Figure 1). The results showed a very good fit of the model to the data (*χ*^2^/df = 1.09; RMSEA = 0.02; SRMR = 0.01; CFI = 0.99). Finally, the mediating effect was tested for significance by adopting the bootstrap estimation procedure (a bootstrap sample of 1,000 was specified). The 95% confidence intervals of the indirect effect of poor sleep quality on occupational well-being through occupational self-efficacy were − 0.08 to −0.20, which did not overlap with zero. The effect of poor sleep quality on occupational well-being through occupational self-efficacy accounted for 26.21% of the total effect. Thus, hypothesis 2 was confirmed and the mediating effect of occupational self-efficacy in the relationship between poor sleep quality and occupational well-being was significant.

## Discussion

This study explored the trilateral relationships among sleep quality, occupational self-efficacy, and occupational well-being. Consistent with the first hypothesis, the results showed that poor sleep quality is significantly and negatively related to occupational self-efficacy and occupational well-being. In support of the second hypothesis, occupational self-efficacy partially mediated the effect of sleep quality on occupational well-being. This study expanded upon existing research on the relationship between sleep quality and well-being in occupational workers, and further verified the mediating mechanism of occupational well-being, which might be of certain theoretical and practical significance.

The results of this study confirmed hypothesis 1 firstly and revealed that poor sleep quality was negatively correlated with occupational well-being. Employees with worse sleep quality tended to be less satisfied with their jobs and experienced less positive affect and more negative affect from their workplaces. These results were consistent with previous studies focusing on other categories of people, such as nurses, fire-fighting officers, and teachers (Arslan et al., 2019). The conservation of resources is among the most important theories that explains occupational well-being (Hobfoll, 2002). This theory holds that individuals are motivated to establish, sustain, and protect four prototypes of resources: physical objects (such as home), personal characteristics (such as well-being), energy (such as knowledge), and conditions (such as marital status). Sleep is an important way to recover psychological resources, including the maintenance of arousal, alertness, attention, memory, and other types of cognitive ability, including emotion-regulating ability (Tanaka and Shirakawa, 2004; Hystad and Eid, 2016; Cheng et al., 2017; Guglielmi et al., 2018). Therefore, sleep is a major source of psychological capital, and low sleep quality causes its deficiency. When psychological capital is insufficient for job requirements, occupational well-being decreases as a result (Sarwar et al., 2021). Hence, sleep quality correlates with occupational well-being.

This study tested hypothesis 2 and found out the mediating role of occupational self-efficacy between sleep quality and occupational well-being. According to the theory of conservation of resources (Hobfoll, 2002), people have the cognitive mechanisms that individuals draw on to positively adjust when their resources are under threat (Hobfoll, 2001; Halbesleben et al., 2014). In line with the point of conservation of resources, occupational self-efficacy is considered a positive cognitive mechanism that helps to safeguard individuals against the negative behavioral and psychological symptoms effects of poor sleep quality and guides their pursuit of positive job affection. Low sleep quality results in a decrease in psychological capital, and self-efficacy is the core component of psychological capital (Hystad and Eid, 2016; Peng et al., 2019). Furthermore, occupational self-efficacy originates from positive feedback obtained from work, and poor sleep quality correlates with decreases in job performance and increases in job-related accidents (Kao et al., 2016). For instance, poor sleep quality leads to mental fatigue, and thereby a decline in alertness, learning, memory, thinking, and executing functions, which together affect work performance (Swanson et al., 2011). Additionally, previous studies have found that sleep quality can significantly predict occupational injury and job accidents (Kao et al., 2016). In other words, poor sleep quality leads to a decrease in job competence and thereby results in the belief of the individual that they are incompetent for the job. Bandura held that self-efficacy could affect the emotion of people while engaging in activities and impact their attitude toward facing difficulties (Bandura, 1993). High self-efficacy made employees more confident in the job, and thus, they had more passion for the job. Moreover, employees with higher self-efficacy were able to resist work pressure and job burnout (Peng et al., 2013; Shoji et al., 2016). In times of difficulty, they were able to adopt positive coping strategies, and hence, experienced a positive emotional status (Tan-Kristanto and Kiropoulos, 2015). In other words, employees with higher occupational self-efficacy had higher occupational well-being.

## Implications

The findings of the current study lend support to the idea that sleep well feel well. Improving the well-being of employees is one of the goals of management (Wright and Cropanzano, 2004). Previous studies have focused on the effects of individual characteristics, job characteristics, and leadership styles on the well-being of employees (Cole et al., 2009; Zhang et al., 2014b). However, the present study demonstrated that sleep quality and occupational self-efficacy significantly correlate with the occupational well-being of employees, which expands the scope of relevant research to some extent. The results indicated that more attention should be paid to poor sleep quality in employees, which impact both the occupational well-being of employees and the realization of organizational goals. In the circumstance that employees experience such poor sleep quality, researchers have integrated and proposed some organizational sleep management strategies (Barnes et al., 2016). For example, job tasks could arrange flexibly and harmoniously, with the restrictions on shift duration and frequency and the arrangement of the work schedule being consistent with the living rhythms of employees. Appropriate sleep hygiene education should be provided, and the basic healthy sleep habits of employees must be cultivated; an example of this is to decrease the pre-sleep use of mobile phones. At the same time, since the mediating effect of occupational self-efficacy in the relationship between poor sleep quality and occupational well-being, occupational self-efficacy training should be adopted for employees, in particular, can promote the anticipated belief of competent for tasks and activities at work, as part of targeted employees’ sense of well-being programs. The process of improving self-efficacy at work may support the development of more job satisfaction and optimistic working affection (Dendinger et al., 2005; Liu, 2019), which, in turn, could help to reduce the effect of poor sleep quality and improve occupational well-being.

## Limitations

This study had some limitations. Firstly, the participants enrolled were junior employees; managers were not involved. Managers and entrepreneurs more frequently suffer from inadequate sleep or low-quality sleep compared with ordinary employees (Luckhaupt et al., 2010). Moreover, low-quality sleep affects the working level of managers, thereby leading to more severe impacts on enterprises. For example, studies have found that following inadequate sleep, leaders were more likely to lose their charm of leadership (Barnes et al., 2016). Furthermore, sleep problems (low-quality sleep and inadequate sleep) may lead to abusive supervision and further decrease the job devotion of subordinates in their teams (Barnes et al., 2015). Hence, the relationship between sleep quality and the occupational well-being of managers should be further explored. Secondly, only one mediating variable (occupational self-efficacy) was considered, which showed that occupational self-efficacy only partially mediated the effect of sleep quality on occupational well-being. Other possible mediating variables (such as self-esteem, coping strategies, emotion regulation, and psychological capital) can be considered in the future. Thirdly, the use of cross-sectional design also faces a series of problems, such as relevant influencing factors cannot be fully considered (e.g., type of job role, stress of role), more importantly, which was inadequate for the causality deduction, indicating that longitudinal studies are needed to further validate the findings. Fourthly, in the current study conducted during the COVID-19 pandemic, the physical and mental state, job satisfaction, and affection of employees may be different from the normal period, which needs further research to confirm this problem and further explicit the relationship among sleep quality, occupational self-efficacy, and well-being.

## Data availability statement

The original contributions presented in the study are included in the article/supplementary material, further inquiries can be directed to the corresponding authors.

## Ethics statement

The research described in this paper met the standards of the Airforce Medical University and has been approved by its Ethics Committee. The patients/participants provided their written informed consent to participate in this study.

## Author contributions

JP, PF, and SW designed the study, performed the data collection, and analyzed the data statistically. JP, JZ, BW, YH, QL, SW, and PF wrote the manuscript. All authors read and approved the final manuscript.

## Funding

This study was funded by the National Natural Science Foundation of China (no. 31900791), the Key project of PLA Logistics Research Program during the 14th Five-Year Plan period (BKJ21J013), the Military Medical Science and Technology Youth Training Program (no. 20QNPY049), and Air Force Medical University “Everest Project” (no. 2020cyjhfp).

## Conflict of interest

The authors declare that the research was conducted in the absence of any commercial or financial relationships that could be construed as a potential conflict of interest.

## Publisher’s note

All claims expressed in this article are solely those of the authors and do not necessarily represent those of their affiliated organizations, or those of the publisher, the editors and the reviewers. Any product that may be evaluated in this article, or claim that may be made by its manufacturer, is not guaranteed or endorsed by the publisher.
